# Spatial and Temporal Variability of Polycyclic Aromatic Hydrocarbons in Sediments from Yellow River-Dominated Margin

**DOI:** 10.1155/2014/654183

**Published:** 2014-10-19

**Authors:** Su Ding, Yunping Xu, Yinghui Wang, Xinyu Zhang, Liang Zhao, Jiaping Ruan, Weichao Wu

**Affiliations:** ^1^MOE Key Laboratory for Earth Surface Processes, College of Urban and Environmental Sciences, Peking University, Beijing 100871, China; ^2^Qingdao Collaborative Innovation Center of Marine Science and Technology, Qingdao 266100, China

## Abstract

Polycyclic aromatic hydrocarbons (PAHs) were analyzed for surface sediments and a sediment core from the Yellow River-dominated margin. The concentration of 16 USEPA priority PAHs in surface sediments ranged from 5.6 to 175.4 ng g^−1^ dry weight sediment (dws) with a mean of 49.1 ng g^−1^ dws. From 1930 to 2011, the distribution of PAHs (37.2 to 210.6 ng g^−1^ dws) was consistent with the socioeconomic development of China. The PAHs' concentration peaked in 1964 and 1986, corresponding to the rapid economic growth in China (1958–1965) and the initiation of the “Reform and Open” policy in 1978, respectively. The applications of molecular diagnostic ratios and principal component analysis suggest that PAHs are predominantly produced by the coal and biomass combustion, whereas the contribution of petroleum combustions slightly increased after the 1970s, synchronous with an increasing usage of oil and gas in China.

## 1. Introduction

Polycyclic aromatic hydrocarbons (PAHs) with two to six rings are a class of organic contaminants mainly derived from incomplete combustion of organic matter, such as coal, fossil fuel, and wood, as well as from forest fires, volcanic activities, and petroleum seeps [1, 2]. PAHs can undergo long-range transport due to the relative recalcitrant and semivolatile properties [3]. Since many PAHs are toxic, carcinogenic, and mutagenic, they have received considerable attention from scientists, governments, and the public [4, 5]. For example, sixteen PAH compounds have been listed as priority pollutants by the United State Environmental Protection Agency (US EPA) as well as the European Union.

Over the past several decades, the fossil fuel use in China has dramatically increased with the rapid economic development [6], resulting in significant increases in PAH loadings to the environment. Such situation is particularly serious in the Bohai Sea surrounding area, one of the biggest economic rims in China [7]. In 2006 alone, 4.8 × 10^9^ tons of wastewater were discharged into the Bohai Sea, causing the failure to meet water quality standards for an area of 2.0 × 10^4^ km^2^ [8]. A number of anthropogenic impact studies focused on PAHs in water, suspended particulate matter and surface sediments in Bohai Sea [7, 9–13]. However, to the best of our knowledge, the spatial variability of PAHs along the transect of the Yellow River to Bohai Sea has not been reported, although the Yellow River is one of the most important conduits in transporting sediments and pollutants from land to sea in northern China [14, 15].

Due to the large sediment load of the Yellow River, the Bohai Sea has an exceptionally high sedimentation rate, and its sediment is an excellent recorder of historical PAHs flux. Several studies reported increasing PAH concentrations during the 20th century in the Yellow Sea, the South China Sea, and the East China Sea [16–19]. Here we measured PAHs in surface sediments and in a sediment core from the lower Yellow River and Bohai Sea. Our main objectives are (1) to evaluate sources of PAHs (e.g., pyrogenic versus petrogenic) in the surface sediments from the Yellow River, estuary, and coast and (2) to assess the relationship between PAHs and local socioeconomic conditions over the past eight decades.

## 2. Material and Methods

### 2.1. Study Area and Sampling

**Figure 1:**
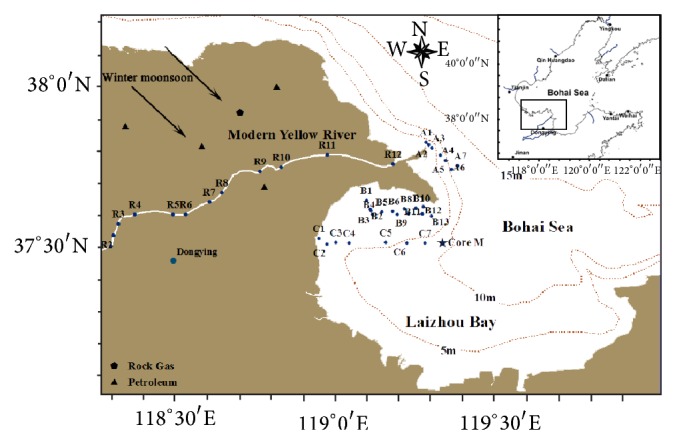
Map of study area and sampling sites in the Yellow River-dominated continental margin. The arrows show prevailing northwesterly wind in winter and spring. Star denotes the site of sediment core.

The Bohai Sea, a shallow, semiclosed sea in northeastern China, has a surface area of 77,000 km^2^ and an average water depth of 18 m [20]. Yellow River, ranking as the world second largest river in term of sediment load [14], has been flowing into the Bohai Sea since 1855 [21, 22]. In July 2011, a total of 39 surface sediment samples (ca. 0–20 cm) were retrieved from the lower Yellow River, estuary, and Laizhou Bay (southern Bohai Sea) by a steel grab sampler, from which the top ~5 cm sediments were picked up by a steel spatula (Figure 1). Due to large terrestrial material inputs, the sedimentation rate is high (>0.8 cm per year) in the Yellow River-dominated margin [23, 24]. Consequently, the top 5 cm sediments represent <7 year sedimentation history. According to geographical locations, the surface samples were classified into four groups, namely, Yellow River (YR) (Sites R1–R12), modern estuary (Sites A1–A7), old estuary (Sites B1–B13), and the coast (Sites C1–C7) (Figure 1). In addition, a 65 cm long piston core (119°18.999′N, 37°30.906′E) was retrieved from Laizhou Bay and was subsectioned on board of ship into 1 cm segments. All samples were transported to the laboratory within 48 hours and frozen at −20°C until analysis. Twelve samples were selected from the sediment core at every 6 cm interval for the PAH analysis.

### 2.2. Sediment Dating

The detailed methods about ^137^Cs and ^210^Pb dating have been reported [23]. The total 40 core samples were used for the radionuclide measurement. About 5 g dried sediments were used for ^137^Cs and ^210^Pb measurements. Counting times of ^137^Cs and ^210^Pb were typically in the range 50,000–86,000 s, giving a measurement precision of between ca. ±5% and ±10% at the 95% level of confidence, respectively. The results showed a mean sedimentation rate of 0.87 cm per year for the upper part (41.5 to 0 cm) and 0.73 cm per year for the lower part (65 to 41.5 cm) [24].

### 2.3. Total Organic Carbon (TOC) Analyses

The sediments were freezing dried at −50°C and grounded into fine powder. After the addition of excess 1 N hydrochloric acid to completely remove inorganic carbon, TOC was measured by an Elementar Vario EL element analyzer. The standard deviation based on the replicate analyses was ±0.02% for TOC.

### 2.4. Sample Extraction and Clean-Up

Freeze-dried sediments (~10 g) were homogenized by a mortar and pestle. After addition of surrogate standards (2-fluorobiphenyl,* p*-terphenyl-d_14_), samples were extracted (3×) with 20 mL* n*-hexane/acetone (1 : 1, v/v) in an ultrasonic bath. The extract was evaporated to 1 mL at 35°C with a rotary evaporator and then fractionated over silica gel/alumina column (30 cm; 10 mm i.d.). The column was first eluted with 25 mL* n*-hexane to remove non-PAHs such as aliphatic hydrocarbons and then with 50 mL mixed dichloromethane and* n*-hexane (1 : 1, v/v) for PAHs. The second fraction was concentrated with the rotary evaporator at 35°C. After addition of 5 mL* n*-hexane, the mixed solution was evaporated to nearly 1 mL, spiked with recovery standards including five deuterated PAHs (naphthalene-d_8_, acenaphthene-d_10_, phenanthrene-d_10_, chrysene-d_12_, and perylene-d_12_), and stored in a 2 mL vial capped with a Teflon-lined septum for further analysis.

### 2.5. Sample Analysis

PAHs were determined on an Agilent 7890A gas chromatography (GC) coupled to a 5975C mass selective detector (MSD). The separation was achieved on a HP-5 MS capillary column (30 m × 0.25 mm i.d. × 0.25 *μ*m film thickness) in a splitless injection mode. The injection temperature was 280°C. The column temperature was programmed from 80°C (held for 1 min) to 270°C at a rate of 5°C min^−1^, then increased to 300°C at a rate of 3°C min^−1^, and held at 300°C for 8 min to purge the chromatographic column. Helium (>99.999%) was used as the carrier gas. The mass spectrometry was operated in the selected ion monitoring (SIM) mode with an electron impact ionization of 70 eV, an ion source of 230°C, and an electron multiplier voltage of 1235 V.

All samples were analyzed for 16 USEPA priority PAHs, including naphthalene (Nap), acenaphthene (Ace), acenaphthylene (Any), fluorine (Fl), phenanthrene (Phe), anthracene (An), fluoranthene (Fla), pyrene (Pyr), benz[a]anthracene (BaA), chrysene (Chr), benzo[b]fluoranthene(BbF), benzo[k]fluoranthene (BkF), benzo[a]pyrene (BaP), indeno[1,2,3-cd]pyrene (IcdP), dibenz[a,h]anthracene (dBA), and benzo[g,h,i]perylene (BPe). Additionally, alkyl PAHs such as 1-methyl phenanthrene (1-MP), 2-methyl phenanthrene (2-MP), 3-methyl phenanthrene (3-MP), and 9-methyl phenanthrene (9-MP) were also measured and quantified according to the respective peak areas. The four methyl phenanthrene isomers were summed and reported as total methyl phenanthrenes (MP).

### 2.6. Quality Assurance and Quality Control

Procedural blanks, spiked blanks, spiked matrixes, and parallel samples were performed to control data quality. As low molecular weight (LMW; 2 + 3 rings) PAHs like Nap and Phe were abundant in the atmosphere, two procedural blanks were added for every ten sample pretreatments. The mean blank value was subtracted from all samples. The detection limits for individual PAH ranged from 0.2 to 2.2 ng g^−1^ dws (dry weight sediment). The recoveries of PAHs in the matrix spiked samples (*n* = 3) ranged from 69.6% (Nap) to 100.5% (BkF), and the recoveries of the surrogates (2-fluorobiphenyl,* p*-terphenyl-d_14_) accounted for 51.9 ± 8.5% and 83.0 ± 11.8% (*n* = 51), respectively (Supplementary Table  1 in Supplementary Material available online at http://dx.doi.org/10.1155/2014/654183). The instrumental precision was determinedby analyzing a standard containing 100 ppb PAHs and 200 ppb deuterated PAHs, and the standard deviation (SD) was less than 5% of the measured values (*n* = 10). Among all sediments, the sample R7 was used for the parallel experiment, and its relative standard deviation (RSD; *n* = 3) was less than 15%, demonstrating that our experimental processes including extraction, clean-up, and GC-MS analysis were well controlled.

### 2.7. Statistical Analysis

To better assess the spatial differences in the PAHs' distribution, a principal component analysis (PCA) was performed (SPSS 17.0, Illinois USA). All 39 samples were included in our PCA. However, two PAHs, Nap and An, were excluded in the PCA because Nap is the most volatile PAH with low environmental stability, while the concentration of An is too low to be quantified in several samples. PAHs were processed on basis of their concentrations (ng g^−1^ dws).

## 3. Results and Discussion

### 3.1. Concentration and Source of PAHs in Surface Sediments

**Figure 2:**
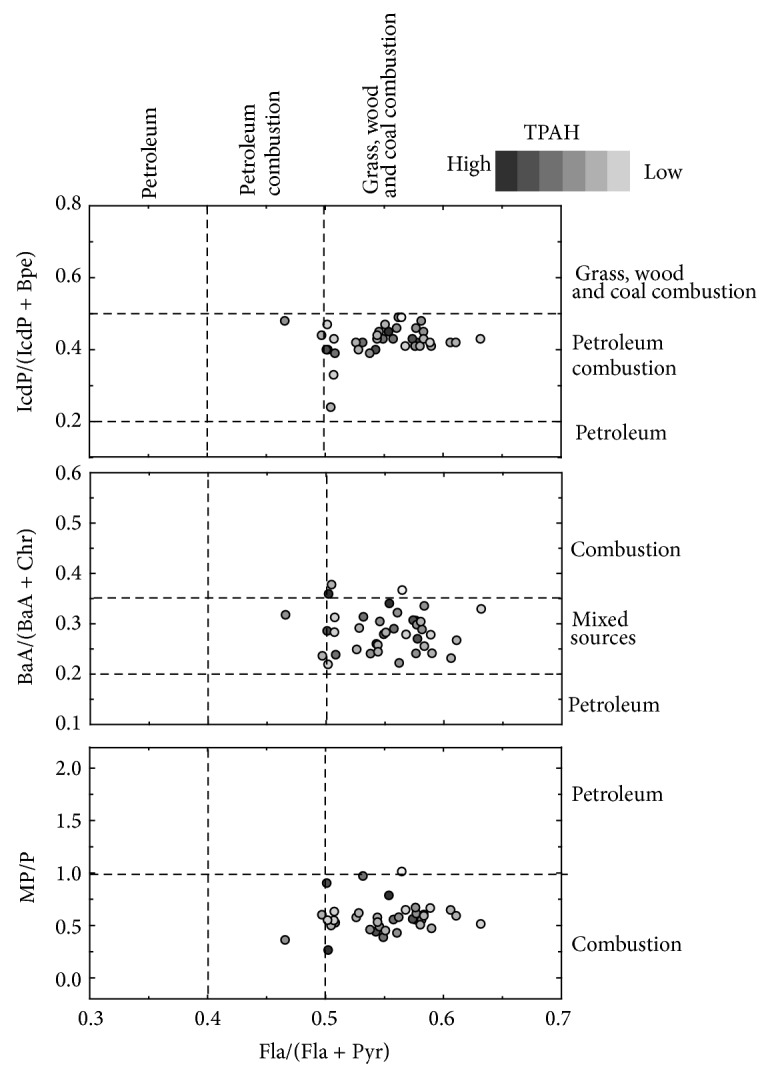
Cross plot of PAHs based on different diagnostic ratios for surface sediments from the Yellow River-dominated continental margin. (a) IcdP/(IcdP + Bpe) versus Fla/(Fla + Pyr); (b) BaA/(BaA + Chr) versus Fla/(Fla + Pyr); (c) MP/P versus Fla/(Fla + Pyr). IcdP: indeno[1,2,3-cd]pyrene; Bpe: benzo[g,h,i]perylene; Fla: fluoranthene; Pyr: pyrene; BaA: benz[a]anthracene; Chr: chrysene; MP: methyl phenanthrenes; P: phenanthrenes. The dash lines represent the thresholds for different sources [1].

The concentration of 16 USEPA priority PAHs (Σ16 PAHs) ranged from 5.6 to 175.4 ng g^−1^ dws with a mean of 49.1 ng g^−1^ dws in our study (Figure 2). Hu et al. [7] and Hui et al. [12] reported a mean Σ16 PAHs of 175.7 ng g^−1^ dws (97.2–300.7 ng g^−1^ dws) and 90.7 ng g^−1^ dws (10.8–252 ng g^−1^ dws), respectively. Therefore, our study has lower PAH abundance compared to previous studies for the Bohai Sea [7, 10–12]. Two factors may explain this difference. First, our samples are from the southern portion of Bohai Sea (Figure 1), further away from Beijing and Tianjin, two megacities near the Bohai Sea with population of over 30 million [10, 11]. Second, the implementation of new energy policy has replaced coal with clean energy resources such as natural gas and hydropower, which reduced the PAH emissions.

Sedimentary TOC can play an important role in PAH accumulation. This was investigated in the present study.  Table 1 shows linear correlations (*r*) of sedimentary TOC contents with different ring PAHs on the basis of 39 surface sediments. Overall, a positive correlation (*r* = 0.54 ~ 0.63; *P* < 0.001) was observed between TOC and PAHs, confirming that sedimentary organic carbon has a somewhat influence on the PAH concentrations in the surface sediments from the Yellow River-dominated margin. The *r* values were the highest for sediments from the coast (>0.95), followed by the old estuary (0.62~0.82), the lower Yellow River (0.57~0.60), and the modern estuary (0.39~0.60). Such difference suggests that an adsorption equilibrium between PAHs and sedimentary TOC has been reached in the coastal area after the long-range transport of terrestrial materials from land to sea, whereas other factors such as mineral size may also contribute to PAH accumulation to some degree in the river and estuaries.

In our study, the PAHs with 2-3 rings, 4 rings, and 5-6 rings accounted for 60 ± 11%, 29 ± 7%, and 11 ± 5% of total PAHs, respectively (Supplementary material Table  2). The predominance of low molecular weight (LMW) over high molecular weight (HMW) PAHs may reflect either preferential inputs of LMW PAHs or an important contribution of petrogenic products or biomass and coal burning at low to moderate temperatures [7, 25]. LMW (2 + 3 rings) PAHs are more volatile and thus are preferentially delivered by atmospheric transport. In addition, LMW PAHs are more abundant in petrogenic products and low to moderate temperature combustion processes (e.g., biomass and coal burning in homes and small factories), whereas HMW (5 + 6 rings) PAHs are products of high temperature combustions involving coal and petroleum such as large power plants and factories, vehicular emissions, and gas-fired cooking operations [26, 27].

PAHs with a petrogenic origin are usually abundant in alkyl PAHs relative to their parent compounds, while those with a pyrogenic (combustion) origin contain little or no alkyl PAHs [28, 29]. In our study, the ratio of alkyl : parent PAHs has a mean value of 0.58 ± 0.17 in the lower Yellow River, 0.60 ± 0.14 in the modern estuary, 0.53 ± 0.11 in the old estuary, and 0.63 ± 0.18 at the coast. The predominance of parent over alkyl PAHs suggests that PAHs in surface sediments of the southern Bohai Sea are mainly produced by pyrogenic processes. One exception is site C7 from the coastal area, which contains more abundant alkyl PAHs than parent PAHs. This suggests an important contribution of petrogenic products probably from oil spills since the Bohai Sea is a busy shipping channel and an important offshore oil base in China [7, 30].

Yunker et al. (2002) proposed that the Fla/(Fla + Pyr) ratio of 0.4-0.5 and >0.5 is indicative of liquid fossil fuel combustion and coal/grass/wood combustion, respectively, whereas the BaA/(BaA + Chr) ratio of 0.2, 0.20–0.35, and >0.35 suggests PAHs primarily from petroleum, mixed petroleum/combustion, and coal/wood combustion, respectively. In addition, the IcdP/(IcdP + Bpe) ratio of <0.2, 0.2–0.5, and >0.5 is characteristic of petroleum, petroleum combustion, and coal/wood combustion, respectively [1]. In surface sediments from the Yellow River-dominated margin, Fla/(Fla + Pyr), BaA/(BaA + Chr), and IcdP/(IcdP + Bpe) vary from 0.47 to 0.63, 0.22 to 0.38, and 0.24 to 0.49, respectively (Figure 2), suggesting that PAHs are mainly produced by the combustions of coal and biomass, while the contribution of liquid fossil fuel combustion is minor [1]. This source apportionment is consistent with the current energy structure of China. It has been reported that over 1.2 billion tons of coal are consumed annually in China, representing 70% of domestic energy consumption [31]. In rural areas of China, wood, grass, and crop-stalk have been widely used for cooking and heating, representing an additional contributor for PAHs [32]. Compared to oil and natural gas, the combustion of coal and biomass releases more PAHs per unit of power generated [33, 34]. Therefore, it is not surprising that the combustion of coal and biomass is the most important source for PAHs in the Yellow River-dominated margin.

### 3.2. Fluvial versus Atmospheric Inputs of PAHs

**Figure 3:**
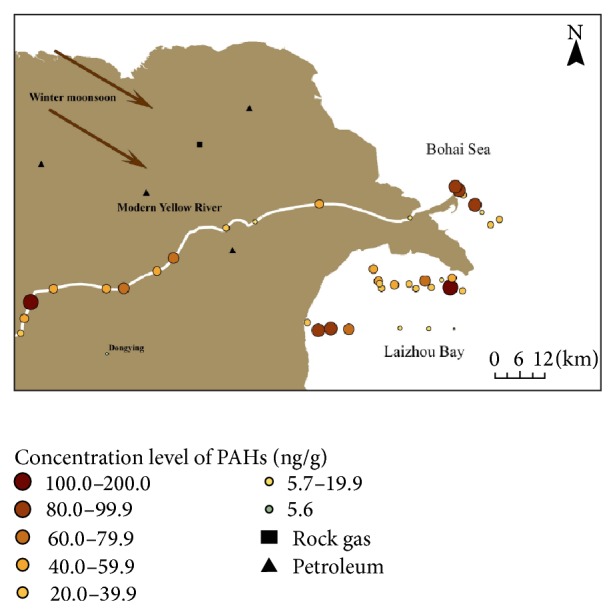
Spatial distributions of PAHs in surface sediments along the transect of lower Yellow River, river mouth, and adjacent sea.

As semivolatile and relatively refractory compounds, PAHs can be transported from land to sea via atmosphere [35]. In winter and spring, the northwesterly winds predominate in the East Asian continent, carrying dust (including PAHs) to the north Pacific [25, 36]. The Bohai Sea is downwind to the Chinese mainland and in the pathway of the outflow East Asian dust in winter and spring (Figure 1). As a result, large amounts of air particles and associated PAHs were deposited in the Bohai Sea [37]. Hafner and Hites [38] observed that the gas-phase reactivity increased with increasing molecular weight of PAHs. Thus, if atmospheric deposition was a sole input pathway for PAHs, relative abundance of HMW PAHs would present a decreasing trend towards sea. Such trend was not observed in our study. From lower Yellow River to the coast, the relative abundance of PAHs with 2-3 rings, 4 rings, and 5-6 rings varied in a narrow range of 59% to 61%, 28% to 30%, and 10% to 12%, respectively (Supplementary material Table  2). The mean Σ16 PAHs was 57.6 ± 46.6 ng g^−1^ dws in the lower Yellow River, 51.7 ± 38.0 ng g^−1^ dws in the modern river estuary, 44.6 ± 26.4 ng g^−1^ dws in the old river estuary, and 45.9 ± 38.9 ng g^−1^ dws at the coast (Figure 3). A statistical analysis shows no significant difference for the PAH concentrations among different sites (*P* > 0.05). These distribution patterns of PAHs suggested the existence of other input pathways besides atmospheric transport.

PAHs are easily adsorbed on surface or occluded inside of suspended particles because of their hydrophobic properties. The particle-associated PAHs can be transported to the sea via runoff [22]. Lin et al. [25] reported riverine transport as a major input pathway of petroleum-derived PAHs in estuarine and coastal areas. In contrast to atmospheric transport, fluvial transport is an enrichment process for HMW PAHs because LMW PAHs are more readily desorbed from sediments in aquatic environments and therefore are more prone to degradation [39, 40]. Given these facts, a homogenous distribution of Σ16 PAHs in surface sediments suggests complex transport mechanisms in the Yellow River-dominated margin.

### 3.3. Principal Components Analysis of PAHs

**Figure 4:**
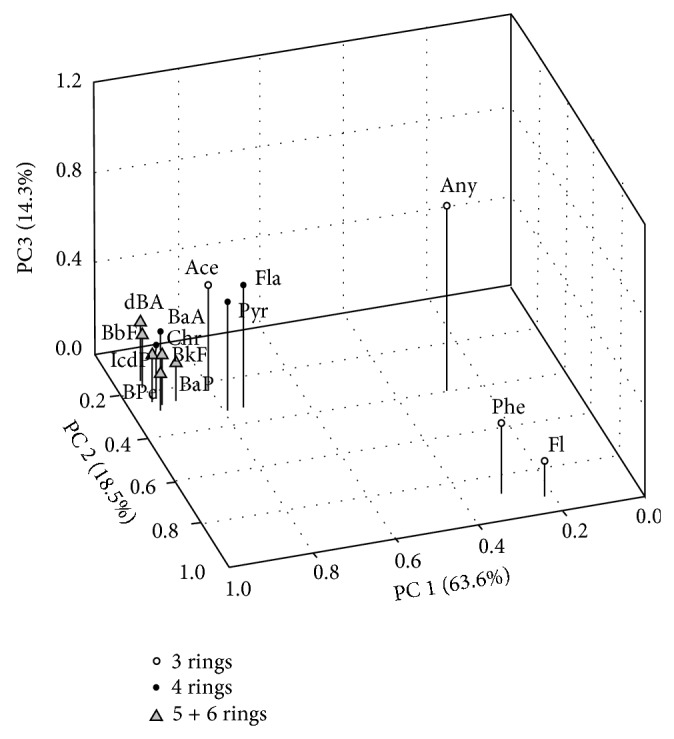
3D loading plot of principal component analysis (PCA) for PAHs in surface sediments from the Yellow River-dominated margin. PC: principal component.

The PCA result shows that the first three principal components (PC) account for 96.5% of total variances (Figure 4). The PC 1 (63.6%) is characterized by high positive loadings of HMW PAHs (>0.75) including BaA, Chr, BkF, BbF, BaP, IcdP, dBA, and BPe. These 4–6 ring PAHs are primarily derived from the high temperature combustion of fossil fuel and biomass [37]. The PC 2 (18.5%) is positively correlated with LMW PAHs including Fl and Phe (loadings of >0.8). These 3 ring PAHs are of a petrogenic origin or produced by the low temperature combustion of coal and biomass [37]. The PC 3 (18.5%) has high positive loadings (>0.48) of Any, Ace, Fla, and Pyr. These 3-4 ring PAHs have mixed pyrogenic/petrogenic sources. Our PCA result demonstrates that PAHs in the surface sediments have a predominant pyrogenic source from the combustion of coal, biomass, and petroleum [27, 41–43], while the inputs from the petrogenic source such as oil spill is minor [44, 45]. The PCA result agrees well with the source apportionment from molecular diagnostic indicators (see Section 3.1).

### 3.4. Temporal Distribution of PAHs

**Figure 5:**
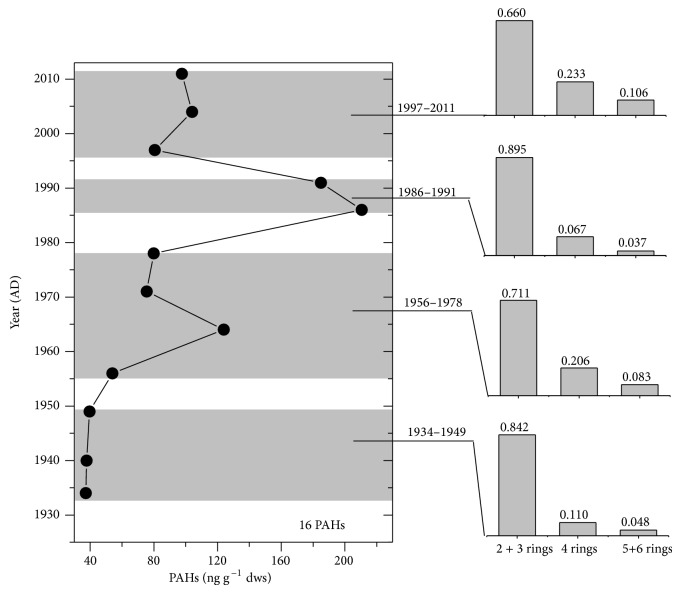
Historical distributions in abundance of total 16 PAHs (ng g^−1^ dws) and percentage of 2 + 3, 4, and 5 + 6 ring PAHs in sediment core from Southern Bohai Sea.

**Figure 6:**
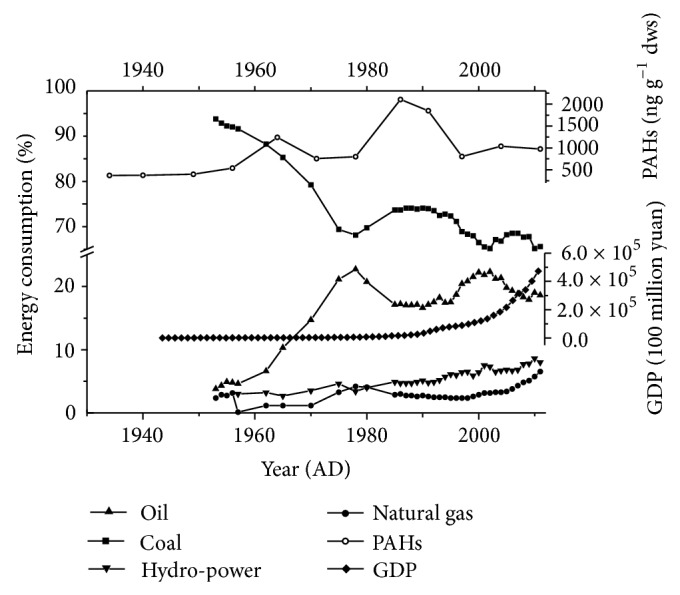
Relationship between sedimentary PAH concentrations (ng g^−1^ dws) in southern Bohai Sea and energy consumption of China.

Figure 5 shows historical variations of Σ16 PAHs (37 to 211 ng g^−1^ dws) in the southern Bohai Sea during the period of ca. 1930–2011. Prior to 1950, the Σ16 PAHs remained lower than 40 ng g^−1^ dws, synchronous with the WWII (1937–1945) and the Chinese Civil War (1946–1949). During this period, many factories moved to inner China from the coastal areas. Our result agrees well with the report by Guo et al. [37] who observed low abundance of Σ16 PAHs from 1937 to 1949 in the East China Sea. Starting in 1950, the Σ16 PAHs increased significantly in the Bohai Sea and reached a maximum in 1964 (124 ng g^−1^ dws). From 1964 to 1978, the Σ16 PAHs declined to 78 ng g^−1^ dws, which is synchronous with the Cultural Revolution in China (1966–1976). During this 10-year period, China was in a long-term recession and many factories were ceased because of political conflicts. A similar decline for PAHs was observed in the South China Sea [16] and the East China Sea [37]. After 1978, the Σ16 PAHs substantially increased to 210 ng g^−1^ dws in 1986 and remained at this high level until early 1990s. This stage with high PAH abundance is consistent with the fast economic development of China after the termination of the Cultural Revolution in 1976 and the initiation of the “Reform and Open Policy” in 1978. However, the Σ16 PAHs decreased to 80 ng g^−1^ dws after 1990 despite a continuous economic growth in China (Figure 6; NBSC, 2012). This decoupling between the economic condition and the PAH abundance is likely induced by the switch of the energy structure in China. Since 1990, increased usage of natural gas and other clean energy resources such as hydropower has caused a decrease in the proportion of coal in the energy consumption (Figure 6) [32]. It is well known that coal releases more PAHs than natural gas and oil per unit of power generated [33, 34]. Similar phenomena have been verified in Europe and the United States as a result of energy structure changes [46, 47].

### 3.5. Historical Variability of PAH Sources

**Figure 7:**
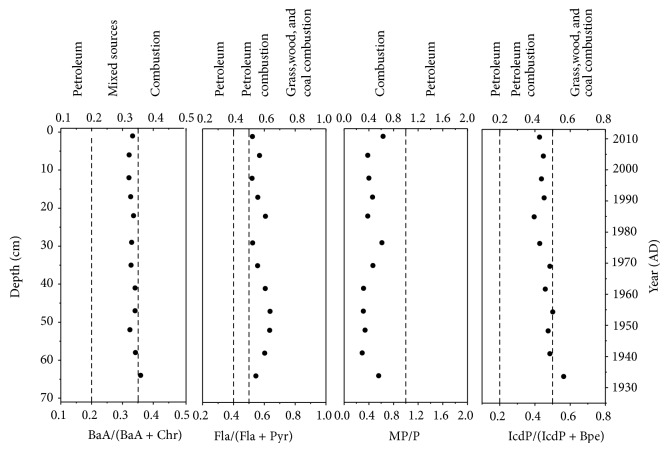
Historical profiles of diagnostic ratios of PAHs in sediment core from southern Bohai Sea, China. IcdP: indeno[1,2,3-cd]pyrene; Bpe: benzo[g,h,i]perylene; Fla: fluoranthene; Pyr: pyrene; BaA: benz[a]anthracene; Chr: chrysene; MP: methyl phenanthrenes; P: phenanthrenes. The dash lines represent the thresholds for different sources [1].

From 1930 to 2011, the relative abundance of 2 + 3 ring PAHs generally decreased (84.2% from 1934 to 1949 versus 66% from 1997 to 2011), whereas the relative abundance of 5 + 6 ring PAHs increased from 4.8% to 10.6% (Figure 5). This shift in the PAH composition suggests more contributions from high temperature combustion of coal and petroleum such as large power plants and factories, vehicular emissions, and gas-fired cooking operations [37]. A series of diagnostic molecular indicators such as BaA/(BaA + Chr), MP/P, and IcdP/(IcdP + Bpe) indicates that PAHs are primarily from the combustion of coal, biomass, and petroleum over the past eight decades (Figure 7). A slight decrease in the Fla/(Fla + Pyr) and IcdP/(IcdP + Bpe) after the 1970s confirms more PAH inputs from the petroleum combustion [1], consistent with an increase in usage of oil and natural gas in the Chinese energy consumption during this period (Figure 7).

## 4. Conclusions

We have examined the spatial and temporal distributions of PAHs in surface sediments and a sediment core from the lower Yellow River, estuary, and Laizhou Bay. Based on data about molecular diagnostic ratios, relative number of aromatic rings, and PCA, four conclusions have been drawn.

(1) Compared to the previous studies, the concentration of 16 USEPA priority PAHs in our study is lower, partially attributed to recent implementation of new energy policy in China. (2) The homogenous distribution of PAHs and the positive relationship between TOC and PAHs' abundance suggest a mixed input by atmospheric and riverine transport. (3) The historical PAHs' pattern in the Laizhou Bay presents a good correlation with the Chinese socioeconomic conditions during the period of 1930 to 2011, confirming that PAHs are a sensitive tracer for anthropogenic activity. (4) The PAHs in the southern Bohai Sea are primarily derived from the combustion of coal and biomass. The contribution of petroleum combustion is minor, but it increased after 1970s, consistent with the change in the Chinese energy structure.

## Supplementary Material

Table S1 presents the recoveries of mixed PAHs in the matrix spiked samples. The recovery was calculated based on three replicate injections on an Agilent GC-MS. Generally, the recoveries varied from 69.7% to 100.5%.
Table S2 shows the concentrations of 16 PAH (ng g^−1^ dws), diagnostic ratios and relative abundance of PAHs with different rings in surface sediments from the Yellow River dominated margin. The total concentration of 16 PAHs varied from 5.6 to 175.6 ng g^−1^ dws with a mean of 49.1 ng g^−1^ dws. The mean percentage of 2 + 3 rings, 4 rings and 5 + 6 rings was 60%, 29% and 11%, respectively. The mean value of Fla/(Fla + Pyr), BaA/(BaA + Chr) and IcdP/(IcdP + Bpe) was 0.55 (0.47~0.63), 0.29 (0.22~0.38) and 0.42 (0.24~0.49), respectively.

## Figures and Tables

**Table 1:** Linear correlations (*R*) of TOC against PAH concentration (ng g^−1^ dws) along the transect of lower Yellow River (YR), river mouth, and adjacent sea.

PAHs	lower YR	Modern estuary	Old estuary	Coast	Entire areas
2 + 3 rings	0.60	0.60	0.62	0.97	0.63
4 rings	0.57	0.39	0.82	0.96	0.53
5 + 6 rings	0.59	0.55	0.75	0.95	0.54

All probability (*P*) values are lower than 0.001. The number of samples (*n*) is 12 for the lower YR, 7 for the old estuary, 13 for the modern estuary, and 7 for the coast.
