# Supplementary material for: Spatial and Temporal Variability of Polycyclic Aromatic Hydrocarbons in Sediments from Yellow River-Dominated Margin
Source: ScientificWorldJournal. 2014 Oct 19;2014:654183. doi: 10.1155/2014/654183 (PMC4217326; doi:10.1155/2014/654183)
Supplement: Supplementary file 1 — Table S1 presents the recoveries of mixed PAHs in the matrix spiked samples. The recovery was calculated based on three replicate injections on an Agilent GC-MS. Generally, the recoveries varied from 69.7% to 100.5%. Table S2 shows the concentrations of 16 PAH (ng g−1 dws), diagnostic ratios and relative abundance of PAHs with different rings in surface sediments from the Yellow River dominated margin. The total concentration of 16 PAHs varied from 5.6 to 175.6 ng g−1 dws with a mean of 49.1 ng g−1 dws. The mean percentage of 2 + 3 rings, 4 rings and 5 + 6 rings was 60%, 29% and 11%, respectively. The mean value of Fla/(Fla + Pyr), BaA/(BaA + Chr) and IcdP/(IcdP + Bpe) was 0.55 (0.47~0.63), 0.29 (0.22~0.38) and 0.42 (0.24~0.49), respectively. [file 654183.f1.pdf]

Table S1: The recoveries of PAHs in the matrix spiked samples (n = 3) ranged from 69.6% (Nap) to 100.5% (BkF)

	Standard added in the matrix*	Spiked matrix PAHs concentrations*			Matrix PAHs concentrations*	Recoveries
		1	2	3		
Nap-d8	20.0	20.0	20.0	20.0	20.0	
Nap	6.2	5.4	4.9	5.8	1.1	69.6%
Ace-d10	20.0	20.0	20.0	20.0	20.0	
ss1	6.8	4.9	4.6	5.9	0.0	75.3%
Any	6.1	5.1	4.2	5.1	0.0	77.8%
Ace	5.9	4.8	4.1	5.0	0.1	77.6%
F	5.6	5.8	4.6	5.5	0.3	88.7%
Phe-d10	20.0	20.0	20.0	20.0	20.0	
Phe	5.7	7.0	5.8	6.5	1.0	95.6%
An	5.8	5.4	4.5	5.1	0.1	85.2%
C1-Phe	5.5	5.3	5.3	5.3	0.2	92.5%
Fl	5.4	5.9	5.3	5.9	0.3	99.0%
Py	5.5	6.0	5.3	6.0	0.3	98.7%
Chr-d12	20.0	20.0	20.0	20.0	20.0	
p-Terp-d14	7.0	7.3	6.7	7.2	0.0	100.5%
BaA	6.6	5.9	5.4	5.3	0.0	83.5%
Chr	5.8	5.7	5.2	5.4	0.1	92.7%
Per-d12	20.0	20.0	20.0	20.0	20.0	
BbF	5.7	5.5	5.2	5.2	0.1	91.4%
BkF	5.0	5.5	4.8	4.8	0.0	100.5%
BaP	6.1	5.6	5.2	5.2	0.0	87.3%
IcdP	5.8	5.1	4.9	4.6	0.1	82.2%
dBA	5.7	5.0	4.7	4.6	0.0	82.6%
BPe	5.4	5.0	4.8	4.7	0.1	87.9%

*the unit of PAHs concentration is ng g-1dws

Table S2: concentrations of 16 PAH (ng g^{-1}dws), diagnostic ratios and relative abundance of PAHs with different rings in surface sediments from the Yellow River dominated margin.

Stations	Latitude	Longitude	$TPAH_{16}$ (ng g^{-1}dws)	Fla/(Fla+Pyr)	BaA/(BaA+Chr)	IcdP/(IcdP+Bpe)	MP/P	Ring size(relative abundance)			TOC%	Concentration of PAH(ng g^{-1}dws)		
								2+3	4	5+6		2+3	4	5+6
lower Yellow River														
R1	118.30	37.51	34.1	0.55	0.28	0.47	0.45	0.50	0.31	0.19	0.094	20.8	7.0	6.4
R2	118.31	37.54	42.9	0.56	0.32	0.46	0.43	0.68	0.22	0.10	0.070	32.8	5.8	4.3
R3	118.33	37.57	175.5	0.55	0.34	0.45	0.79	0.41	0.38	0.21	0.240	92.5	45.6	37.4
R4	118.38	37.60	47.4	0.47	0.32	0.48	0.36	0.75	0.21	0.04	0.031	39.8	5.8	1.8
R5	118.49	37.60	46.2	0.54	0.24	0.39	0.46	0.70	0.20	0.10	0.290	35.4	6.3	4.5
R6	118.53	37.60	73.7	0.51	0.24	0.39	0.00	0.60	0.25	0.16	0.186	49.6	12.7	11.4
R7	118.61	37.64	44.7	0.58	0.30	0.46	0.61	0.59	0.31	0.10	0.096	32.0	8.3	4.4
R8	118.64	37.67	79.9	0.53	0.31	0.42	0.97	0.43	0.40	0.17	0.162	44.3	21.8	13.8
R9	118.76	37.74	22.5	0.50	0.38	0.24	0.50	0.74	0.22	0.04	0.068	18.9	2.8	0.9
R10	118.82	37.75	16.9	0.50	0.22	0.47	0.55	0.54	0.33	0.13	0.104	11.3	3.5	2.1
R11	118.97	37.79	51.3	0.58	0.34	0.45	0.60	0.54	0.33	0.12	0.132	34.3	10.7	6.3
R12	119.17	37.76	17.5	0.59	0.28	0.42	0.67	0.77	0.18	0.05	0.034	15.0	1.7	0.8
Modern estuaries														
A1	119.27	37.83	89.1	0.50	0.29	0.40	0.90	0.36	0.43	0.21	0.073	42.8	27.4	18.9
A2	119.28	37.82	89.9	0.58	0.31	0.41	0.56	0.76	0.22	0.02	0.076	78.2	9.9	1.7

A3	119.28	37.81	20.4	0.54	0.26	0.43	0.58	0.59	0.29	0.12	0.030	14.2	3.8	2.4
A4	119.31	37.79	97.6	0.54	0.26	0.40	0.44	0.62	0.24	0.14	0.282	68.8	15.2	13.6
A5	119.33	37.77	19.3	0.63	0.33	0.43	0.51	0.79	0.19	0.02	0.021	17.4	1.5	0.3
A6	119.34	37.75	21.3	0.50	0.24	0.44	0.60	0.60	0.38	0.02	0.030	16.6	4.4	0.4
A7	119.36	37.76	24.3	0.53	0.25	0.42	0.58	0.54	0.31	0.15	0.109	15.5	5.3	3.6
Old estuaries														
B1	119.09	37.65	45.2	0.56	0.22	0.49	0.58	0.52	0.33	0.15	0.170	28.9	9.8	6.6
B2	119.10	37.62	53.4	0.58	0.24	0.41	0.67	0.48	0.37	0.15	0.345	33.3	12.2	7.9
B3	119.10	37.61	38.5	0.54	0.24	0.44	0.53	0.47	0.37	0.16	0.141	23.0	9.2	6.3
B4	119.10	37.60	39.2	0.61	0.27	0.42	0.59	0.54	0.35	0.12	0.157	26.8	7.8	4.6
B5	119.13	37.61	47.7	0.58	0.29	0.48	0.55	0.49	0.34	0.17	0.127	29.2	10.2	8.3
B6	119.16	37.61	36.1	0.61	0.23	0.42	0.65	0.61	0.30	0.09	0.142	27.1	5.8	3.2
B7	119.18	37.60	25.4	0.58	0.26	0.43	0.59	0.58	0.31	0.10	0.119	18.1	4.7	2.6
B8	119.20	37.62	76.5	0.55	0.28	0.43	0.39	0.64	0.25	0.11	0.172	55.2	12.8	8.5
B9	119.21	37.61	31	0.53	0.29	0.40	0.62	0.49	0.34	0.16	0.095	18.7	7.2	5.1
B10	119.24	37.62	8.1	0.51	0.28	0.33	0.55	0.58	0.37	0.05	0.030	6.0	1.7	0.4
B11	119.26	37.61	113.5	0.50	0.36	0.40	0.27	0.72	0.18	0.10	0.260	88.0	14.7	10.8
B12	119.26	37.63	42.4	0.55	0.30	0.45	0.49	0.72	0.21	0.07	0.113	34.1	5.5	2.9
B13	119.28	37.60	22.2	0.58	0.30	0.41	0.51	0.78	0.17	0.05	0.037	19.0	2.1	1.1
Coast														
C1	118.94	37.53	20.5	0.59	0.24	0.41	0.47	0.56	0.28	0.17	0.083	13.3	3.8	3.4
C2	118.96	37.51	98.9	0.57	0.31	0.43	0.56	0.62	0.29	0.10	0.253	72.6	16.8	9.5
C3	118.99	37.52	83.8	0.58	0.27	0.42	0.56	0.59	0.29	0.12	0.275	58.2	15.3	10.2
C4	119.03	37.51	77	0.56	0.29	0.43	0.56	0.55	0.31	0.14	0.195	50.5	15.9	10.6

C5	119.14	37.52	18.6	0.57	0.28	0.41	0.65	0.58	0.31	0.11	0.078	13.2	3.4	2.0
C6	119.21	37.52	16.7	0.51	0.31	0.43	0.63	0.66	0.26	0.09	0.053	12.5	2.7	1.4
C7	119.26	37.52	5.6	0.56	0.37	0.49	1.01	0.54	0.36	0.10	0.033	4.1	1.0	0.6
